# Calcium-activated chloride channel regulator 1 as a prognostic biomarker in pancreatic ductal adenocarcinoma

**DOI:** 10.1186/s12885-018-5013-2

**Published:** 2018-11-12

**Authors:** Dingyuan Hu, Daniel Ansari, Qimin Zhou, Agata Sasor, Katarzyna Said Hilmersson, Monika Bauden, Yi Jiang, Roland Andersson

**Affiliations:** 1 Department of Surgery, Clinical Sciences Lund, Lund University and Skåne University Hospital, SE-221 85 Lund, Sweden; 2 Department of Gastroenterology, The Second Affiliated Hospital and Yuying Children’s Hospital of Wenzhou Medical University, Wenzhou, 325017 China; 3 Department of Pathology, Skåne University Hospital, 221 85 Lund, Sweden

**Keywords:** Pancreatic ductal adenocarcinoma, CLCA1, Calcium-activated chloride channel regulators, Survival

## Abstract

**Background:**

In a previous study utilizing mass spectrometry-based proteomics, we identified calcium-activated chloride channel regulator 1 (CLCA1) as a potential tumor suppressor in pancreatic cancer and the expression was inversely correlated with patient survival. The aim of the study was to further validate the prognostic significance of CLCA1 in pancreatic cancer.

**Methods:**

CLCA1 expression was evaluated with tissue microarrays and immunohistochemistry in 140 patients with pancreatic ductal adenocarcinoma that underwent surgical resection at Skåne University Hospital, Sweden. Kaplan-Meier and Cox proportional hazards modeling were used to explore the association between CLCA1 and clinicopathological factors and survival.

**Results:**

CLCA1 expression was denoted as positive in 90 tumors (64.3%), with positive staining being limited to the tumor cells. There were no significant association between CLCA1 expression and established clinicopathological parameters. Low CLCA1 expression correlated significantly with shorter disease-free survival (11.9 vs 17.5 months, *P* = 0.042). Multivariable Cox regression analysis confirmed the results (HR 0.61, 95% CI-0.40-0.92, *P* = 0.019).

**Conclusions:**

Low CLCA1 expression is an independent factor of poor disease-free survival in pancreatic cancer.

## Background

Pancreatic ductal adenocarcinoma (PDAC) is currently the third leading cause of cancer-related mortality [1]. Although achievements have been made to improve the diagnosis and treatment of PDAC, the five-year survival rate remains as low as 6% [2]. Due to the silent progression of the disease and lack of early screening techniques, most patients are diagnosed at an advanced stage, precluding potentially curative surgery. Moreover, tumor heterogeneity is strongly implicated in the biological behavior of PDAC, as well as the response to therapy [3]. More information is needed concerning molecular factors that can contribute to an earlier diagnosis and a better prediction of prognosis and treatment response.

Calcium-activated chloride channel regulators (CLCAs), also called “chloride channel accessory proteins”, are a family of secreted self-cleaving proteins which activate calcium-dependent chloride currents. The human genome encodes 3 functional CLCA proteins, including CLCA1, CLCA2, and CLCA4. As one form of ion channels, Ca^2+^-activated chloride channels have been implicated in regulation of cell proliferation, cell migration and metastasis and are believed to be emerging therapeutic targets in cancer [4–6]. CLCA1 is mainly expressed in the large and small intestine and appendix, especially in crypt cells, and can be shed into the blood stream. It has been reported that CLCA1 can regulate the differentiation of colorectal cancer cells and function as a prognostic marker in colorectal cancer [7]. Recent studies also supported that CLCA2 and CLCA4 may serve as tumor suppressors in breast cancer [8].

Our previous mass spectrometry-based proteomics study showed for the first time that CLCA1 is a biomarker for PDAC, with protein expression being 20 fold down-regulated in poor outcome PDAC patients [9]. The aim of the present study was to investigate the prognostic impact of CLCA1 in a large and well annotated clinical cohort of resectable PDAC patients.

## Methods

### Patients and samples

The REMARK guidelines were followed where possible throughout the whole study [10]. Formalin-fixed paraffin-embedded tissue samples were collected from 140 patients with PDAC who underwent pancreatic resection at the Department of Surgery, Skåne University Hospital, Lund and Malmö, Sweden, between 1996 and 2017. All tissue specimens were re-evaluated by a senior pancreas pathologist (A.S.) to ensure correct diagnosis and histopathological characterization. Disease-free survival (DFS) was defined as the time from pancreatectomy to the first evidence of clinical recurrence (locoregional or distant) or death from any cause. Overall survival (OS) was defined as the time from pancreatectomy to death from any cause or the last date the patient was seen alive. Ethical approval was obtained from the local human ethics committee at Lund University (Ref 2017/320).

### Tissue microarray

Tumors with sufficient amount of material were deemed suitable for tissue microarray (TMA) construction. Compared with usage of whole sections, TMA has the advantage of a reduced consumption of both tissue and time which enables studies of a larger scale with reduced experimental variability [11]. Using an automated tissue arraying device (Minicore® 3, Alphelys, Plaisir, France), 4 cores ⌀ 2 mm of cancer tissues (marked by pathologist A.S) from each specimen were stabilized into paraffin blocks. After a fine quality was assured, the TMA-blocks were sectioned for immunohistochemical analysis.

### Immunohistochemistry

TMA-sections (3 μm thick) were heated in 60 °C for 1 h and then cooled in room temperature (RT). Next, using automated PT Link (Dako, Glostrup, Denmark), deparaffinization, rehydration and antigen-retrieval were performed in EnVision FLEX Target Retrieval Solution high pH (K800421–2, Dako) heated to 96 °C for 20 min. After three times of wash in phosphate-buffered saline for 5 min, sections were blocked against endogenous peroxidase activity with 0.3% H_2_0_2_ and 1% methanol in phosphate-buffered saline for 10 min. The specimens were then blocked with 5% goat serum for 1 h at RT to reduce non-specific background staining, followed by avidin/biotin blocking kit (SP-2001, Vector Laboratories, Burlingame, CA, USA) for 15 min at RT, which reduces endogenous avidin and biotin activity. Subsequently, the sections were incubated with rabbit recombinant monoclonal CLCA1 antibody (Abcam, Cambridge, UK; cat no ab180851; dilution 1:2000) at 4 °C overnight. Next, sections were incubated with biotinylated secondary goat anti-rabbit antibodies (BA-1000, dilution 1:200, Vector Laboratories) for 1 h at RT. Following incubation with avidin-biotin-peroxidase complex (Vectastain Elite ABC-HRP Kit, PK-6100, Vector Laboratories) for 30 min at RT, the sections were incubated with chromogen diaminobenzidine (SK-4100, Vector Laboratories) for 5 min. After washing in deionized water for 5 min, nuclei were counterstained with Mayer’s hematoxylin (Histolab, Gothenburg, Sweden) for 30 s, and washed in tap water for another 5 min. Finally, the specimens were dehydrated in graded alcohols and mounted using Pertex (Histolab). Negative controls were produced by omitting the primary antibodies. Slides were scanned for evaluation using an Aperio scanscope scanner (Leica Biosystems, Wetzlar, Germany). Variability between individuals was reduced by limiting sample preparation and analysis of expression to one professional respectively.

### Scoring procedure

The immunostaining of CLCA1 was assessed semi-quantitatively by an experienced pancreas pathologist (A.S.) blinded to the clinical outcome. Staining below 10% was denoted as negative (0). When > 10% of tumor cells were stained, expression was considered positive and denoted as mild (1), moderate (2) or strong (3) depending on the intensity. Samples with negative staining (0) and mild staining (1) were categorized as low expression group, while those with moderate (2) and strong (3) were categorized as high expression group.

### Statistical analysis

Comparisons of categorical data were performed using Chi-square test or Fisher’s exact test. Continuous data were compared by the Mann Whitney U test. Kaplan–Meier analysis and log rank test were used to illustrate differences in DFS and OS according to CLCA1 expression. Cox regression proportional hazards models were used for estimation of hazard ratios (HRs) for recurrence and death according to CLCA1 expression. Any variable with a *P*-value less than 0.25 was selected as a candidate for the mulvariable Cox regression analysis. In the iterative process of variable selection using forward, backward and stepwise selection covariates were removed from the model if they were non-significant and not a confounder as described by Hosmer-Lemeshow, resulting in the main effect model [12]. A *P*-value less than 0.05 was considered statistically significant. All the statistics were performed using STATA MP 14.1.

## Results

### Patient cohort

Baseline characteristics of patients with PDAC are presented in Table 1. The median age was 69 years (interquartile range 63–73 years) and 73 (52.1%) were female. The estimated median DFS was 13.2 months and the estimated median OS was 25.0 months, respectively. One hundred thirteen (80.7%) of patients received adjuvant chemotherapy.

**Table 1:** Clinicopathological characteristics of patients with pancreatic ductal adenocarcinoma stratified by CLCA1 expression

Factors	Low CLCA1 *N* = 87	High CLCA1 *N* = 53	*P* value	Missing
Age, years	69 (62–75)	68 (64–72)	0.258	
Female gender	44 (50.6)	29 (54.7)	0.634	
T-stage			0.649	0.7%
- T1	12 (13.8)	7 (13.2)		
- T2	58 (66.7)	35 (66.0)		
- T3	16 (18.4)	10 (18.9)		
- T4	0	1 (1.9)		
N-stage			0.485	1.4%
- N0	21 (24.1)	12 (22.6)		
- N1	30 (34.5)	24 (45.3)		
- N2	34 (39.1)	17 (32.1)		
AJCC stage, 8th edition			0.835	1.4%
- IA	4 (4.6)	2 (3.8)		
- IB	12 (13.8)	7 (13.2)		
- IIA	4 (4.6)	3 (5.7)		
- IIB	31 (35.6)	23 (43.4)		
- III	34 (39.1)	18 (34.0)		
Tumor differentiation			0.879	1.4%
- Well	4 (4.6)	3 (5.7)		
- Moderate	28 (32.2)	20 (37.7)		
- Poor	50 (57.5)	29 (54.7)		
- Undifferentiated	3 (3.4)	1 (1.9)		
R1 resection margin	35 (40.2)	20 (37.7)	0.552	0.7%
Adjuvant chemotherapy	68 (78.2)	45 (84.9)	0.129	3.6%

Qualitative data are expressed as N (%) and quantitative data as median (interquartile range). *AJCC* American joint committee on cancer

### CLCA1 expression in PDAC

**Fig. 1:**
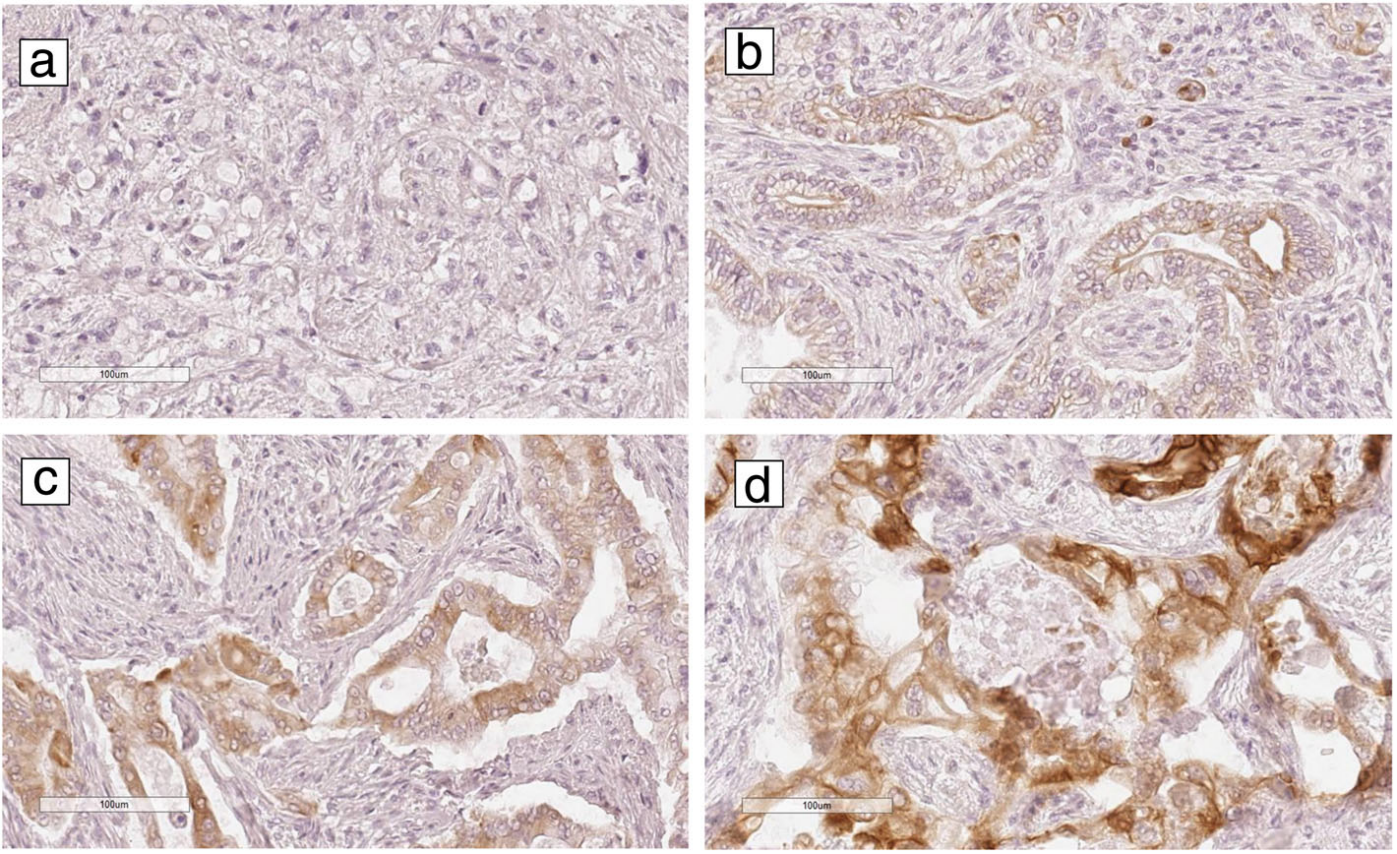
Representative immunohistochemical images of CLCA1 expression in pancreatic ductal adenocarcinoma. **a** negative, **b** weak, **c** moderate, **d** strong staining

CLCA1 expression was considered positive in 90 (64.3%) of the 140 tumors. The expression of CLCA1 was limited to the tumor cells. Mild, moderate and strong staining of CLCA1 were present in 37 (26.4%), 41 (29.3%) and 12 (8.6%) cases respectively. Figure 1 shows representative immunohistochemical images of CLCA1 expression in PDAC.

### Association between CLCA1 expression and clinicopathological characteristics

The expression of CLCA1 was not associated with any traditional clinical parameters, including age, gender, TNM stage, histological grade, resection margin status and adjuvant chemotherapy.

### Association between CLCA1 expression and survival

**Fig. 2:**
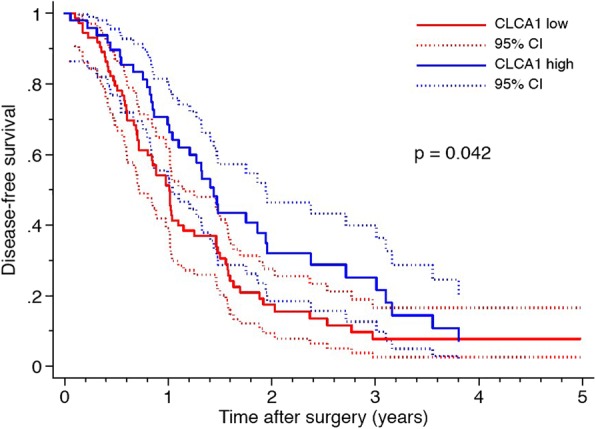
Low expression of CLCA1 is associated with a poor DFS in pancreatic cancer patients undergoing surgical resection

**Fig. 3:**
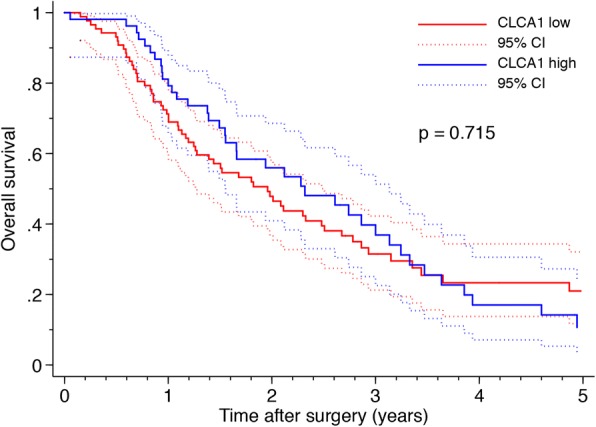
Association between CLCA1 expression and OS in pancreatic cancer patients undergoing surgical resection

Kaplan–Meier analysis revealed that CLCA1 expression correlated with a significantly shorter DFS, with the worst outcome for tumors with low CLCA1 expression (Fig. 2). Median DFS was 11.9 months in patients with low CLCA1 expression and 17.5 months in patients with high CLCA1 expression, *P* = 0.042. These findings were confirmed in univariable Cox regression analysis (HR 0.66, 95% CI-0.44-0.99, *P* = 0.044), and remained significant in multivariable analysis (HR 0.61, 95% CI-0.40-0.92, *P* = 0.019), adjusted for differentiation grade and resection margin status (Table 2). The OS was also reduced in patients with low CLCA1, but the association did not reach statistical significance. The median OS was 23.5 months in patients with low CLCA1 expression and 27.8 months in patients with high CLCA1 expression (*P* > 0.05) (Fig. 3).

**Table 2:** Univariable and multivariable Cox survival analyses for DFS

Variables	Univariable analysis			Multivariable analysis		
	HR	95%CI	*P* value	HR	95% CI	*P* value
Age	0.98	0.96–1.00	0.076			
Female gender	0.66	0.45–0.98	0.039			
T-stage	1.14	0.82–1.58	0.441			
N-stage	1.14	0.89–1.45	0.311			
Differentiation grade	1.48	1.06–2.07	0.023	1.55	1.09–2.18	0.014
Resection margin (R1)	1.55	1.02–2.33	0.036	1.63	1.07–2.49	0.023
Adjuvant chemotherapy	1.57	0.86–2.89	0.144			
CLCA1 expression, high vs low	0.66	0.44–0.99	0.044	0.61	0.40–0.92	0.019

## Discussion

This study demonstrated that low CLCA1 expression is an independent factor of shorter DFS. This is in line with our previous proteomics work utilizing mass spectrometry [9], validating our findings with an orthogonal technique in a larger cohort.

Ion channels, in general, and Ca^2+^-activated chloride channels in particular, are known to be involved in the regulation of cell proliferation, cell migration and metastasis and are considered emerging cancer drug targets [5, 6]. Several studies have reported that the CLCA1 expression is down-regulated in colorectal cancer tissues compared with adjacent normal tissues [13–16], with low CLCA1 expression predicting worse outcomes [7, 17]. Knockdown of CLCA1 in Caco-2 cell lines have been shown to inhibit cell differentiation and promote cell proliferation [15]. Further in-vitro experiments suggested that CLCA1 may function as a tumor suppressor in colorectal cancer by inhibiting the Wnt/beta-catenin signaling pathway and epithelial-mesenchymal transition, while in-vivo overexpression of CLCA1 led to inhibition of proliferation and metastasis [14].

However, there is scant literature on the expression pattern and underlying function of CLCA1 in PDAC. Protein expression data in the Human Protein Atlas indicate that CLCA1 is mainly expressed in small and large intestines and appendix, while absent in both normal and cancerous pancreas tissues [18]. However, we noted that CLCA1 was present in more than half of pancreatic cancer tissues in our study. It is worth mentioning that CLCA1 can be secreted into pancreatic cyst fluid and the blood stream, which makes the CLCA1 a possible serum and fluid biomarker for PDAC [19]. Most recent evidence supported that CLCA1 mediates metalloprotease activity and is involved in intestinal mucus homeostasis by facilitating processing and removal of mucus [20]. This arises interest to address whether similar mechanisms are implicated in intraductal papillary mucinous neoplasm, a precursor of PDAC manifested with mucus contained cyst. Indeed, CLCA1 has been proposed as a supportive marker for high-grade dysplasia and malignant transformation using cyst fluid samples [19].

PDAC is characterized by a dense and heterogenous tumor microenvironment (TME), which drives tumor progression and resistance to therapy. While the past four decades have seen no decline in death rates of this devastating malignancy [1], a better understanding of the mechanisms how pancreatic cancer cell interactions with their TME might open new avenues of research in effective treatments of PDAC. Ion channels are involved in intracellular signaling events and activate specific cellular responses, including cancer-related proliferation, apoptosis, migration and angiogenesis [21]. Ion channels and their interactions with integrin in TME can contribute to tumor development and emerging drug targets [22]. For example, neutrophils in the TME release Cl^−^ to accomplish their antimicrobial activity [23]. Furthermore, activated vascular endothelial cells are required for angiogenesis, in which Ca^2+^ permeable channels and Ca^2+^-dependent signaling play crucial roles [21]. Abdel-Gany et al. also confirmed that CLCAs facilitated vascular arrest of cancer cells via interacting with β_4_ integrin and promote metastatic growth [23].

Secreted CLCA1 has been demonstrated to be a direct modulator of another calcium-dependent chloride channel, TMEM16A [24, 25]. CLCA1 can stabilize TMEM16A on the cell surface and prevent its internalization, thus activating chloride currents [24, 25]. While the role of CLCA1 in PDAC remain unclear, TMEM16A was found to be overexpressed in PDAC cells and promote the cell migration [26]. TMEM16A has also been proposed to contribute to tumor growth and invasion of lung cancer, prostate cancer and head and neck squamous cell carcinomas [27–29].

In this study, CLCA1 predicted DFS, but not OS. Although DFS and OS are partly related, there are differences. In DFS, any type of recurrence or spread is counted as an event, including isolated local recurrences. Patients with low CLCA1 expression seemed to have more early recurrences, i.e. occurring within 1 year of surgery. Multimodal treatment of recurrent pancreatic cancer has been found to prolong survival [30, 31]. However, after 5 years of median follow-up, the number at risk in the patient cohort was only 11 patients due to the high mortality rate. Therefore, our sample size might be underpowered to show a statistically meaningful result in terms of OS.

## Conclusion

This study shows that low CLCA1 expression is a predictor of worse DFS in PDAC. CLCA1 may in the future be integrated into an immunohistochemistry panel to predict prognosis and treatment response in patients who undergo surgical resection. As ion channels have been suggested as emerging cancer drug targets, further investigation into the molecular mechanisms of CLCA1 in PDAC is needed.

## Abbreviations

CLCA1: Calcium-activated chloride channel regulator 1
DFS: Disease-free survival
HRs: Hazard ratios
OS: Overall survival
PDAC: Pancreatic ductal adenocarcinoma
RT: Room temperature
TMA: Tissue microarray
TME: Tumor microenvironment
